# From disobedience to struggle for adaptation: ​​nursing students’ experiences of attending the clinical learning environment during Covid-19 pandemic

**DOI:** 10.1186/s12888-023-04807-8

**Published:** 2023-05-02

**Authors:** Zeinab Jokar, Camellia Torabizadeh, Mahnaz Rakhshan, Majid Najafi Kalyani

**Affiliations:** 1 Student Research Committee, School of Nursing and Midwifery, Shiraz University of Medical Sciences, Shiraz, Iran; 2 Community Based Psychiatric Care Research Center, Department of Medical Surgical Nursing, School of Nursing and Midwifery, Shiraz University of Medical Sciences, Shiraz, Iran; 3 Department of Medical Surgical Nursing, School of Nursing and Midwifery, Shiraz University of Medical Sciences, Shiraz, Iran; 4 Department of Medical Emergencies, School of Nursing and Midwifery, Shiraz Univesity of Medical Sciences, Shiraz, Iran

**Keywords:** Clinical learning environment, Nursing students, Covid-19, Adaptation, Nursing

## Abstract

**Background:**

The Covid-19 pandemic has affected the clinical education of nursing students all over the world. Considering the importance of clinical education and clinical learning environment (CLE) in the education of nursing students, identifying the challenges and problems faced by these students during the covid-19 pandemic helps to plan more effectively in this regard. The aim of this study was to investigate the experiences of nursing students in CLEs during the Covid-19 pandemic.

**Methods:**

A descriptive qualitative research was conducted, which used a purposive sampling technique to recruit 15 undergraduate nursing students from Shiraz University of Medical Sciences between July 2021 and September 2022. Data were collected through in-depth semi-structured interviews. For data analysis, conventional qualitative content analysis based on Graneheim and Lundman approach was used.

**Results:**

The data analysis led to emergence of two themes of “disobedience " and “struggle for adaptation”. The disobedience theme consists of two categories: “objection to attend CLE” and “patient marginalization”. The theme of struggle for adaptation includes two categories: “using support sources” and “applying problem-oriented strategies”.

**Conclusion:**

At the beginning of the pandemic, the students were unfamiliar due to the disease, as well as fear of contracting themselves and infecting others, so they tried not to be in the clinical environment. However, they gradually tried to adapt to the existing conditions by applying support resources and using problem-oriented strategies. Policymakers and educational planners can use the results of this study to plan for solving the challenges of students during future pandemics and improve the condition of CLE.

## Background

Nursing education includes theoretical and clinical education [1]. Clinical education plays an important role in acquiring the professional competences of nursing students because of combining theoretical learning with practical skills [2]. Clinical education plays an important role in personal development and professional competences as well as the improvement of clinical skills of students. As such, many experts consider it as the heart, essence, and integral part of nursing education and the most important factor for learning in nursing environment [3, 4]. Clinical education provides students with patient care-related information and skills [4]. The successful patient care largely depends on an efficient clinical education [1]. Also, clinical education helps students to reflect on their learning [2].

Clinical education mostly takes place in a complex CLE [5]. CLE provides an opportunity for students to learn experientially and transform their theoretical knowledge into various psychomotor skills required for patient care through interaction with patients, nurses, and other healthcare members in a real environment [3, 5]. CLE also enhances creativity and innovation since students are exposed to special and exceptional conditions [6, 7].

In addition to familiarizing students with existing realities, CLEs can cause challenges and problems due to their unpredictable nature [1, 5]. One of the realities that nursing students have faced since 2019 is the Covid-19 pandemic, which has created more and new challenges, problems, and risks for students [8, 9].

The Covid-19 pandemic has affected all areas, including nursing education, in such a way that face-to-face education has become limited or suspended [10–13]. Despite existing changes in patient care and their impact on educational opportunities, students must be present in CLEs [14]. Clinical education is a basic need for medical students, especially nursing students, and cannot be replaced by other methods; thus, face-to-face clinical education is necessary for nursing students during the Covid-19 pandemic [12].

Online CLE not only makes it difficult for students to return to face-to-face CLE environment, but also potentially affects the appraisal of students’ competence by instructors, patients, and even the students themselves [15]. The closure of clinical education is disastrous for nursing students and can cause students to worry about their poor clinical skills and the uncertainty of when, where, and how this educational interruption will be compensated, which in turn causes more stress [16]. Silva et al. showed that midwifery students could not provide health care services to patients due to the cessation of face-to-face clinical education during the Covid-19 pandemic, which in turn leads to negative economic consequences and a misunderstanding about the university administration [17]. In their study, Dos Santos et al. found that almost all participating students decided to leave the nursing profession due to the Covid-19 pandemic [18]. The occurrence of such pandemics and other crises creates valuable and relatively rare clinical education experiences for students as they may face similar pandemics in their profession in the future; therefore, being aware and responsive to current threats such as Covid-19 can increase their knowledge and ability in future crises as well [19]. Further, the experiences gained during critical situations can affect students’ professional development and patient care procedures [14].

The nursing students who were active in CLE during the pandemic considered themselves a part of history and stated that they learned new things and took a step into a valuable challenge in terms of career and personality [8]. Although students are at the front line of the fight against Covid-19, there is little evidence of formal support programs during the transition from a student nurse to a full-time professional nurse [20]. Although this voluntary experience is very helpful to compensate part of the needs of hospitals and will be effective in the development of human character, altruism and work experience of students; there is the fear that this early transition will cause the birth of vulnerable nurses [21].

Previous domestic and foreign studies show that insufficient attention is paid to the entry of students into CLE during the covid-19 pandemic, and a literature review indicates very limited studies on experiences of nursing students in CLEs during the covid-19 pandemic [17, 18, 22, 23]. Moreover, most of these studies have been quantitative and based on predetermined knowledge, and there are few qualitative studies on people’s beliefs and values.

In Iran, with the spread of the COVID-19 and the sudden closure of universities, nursing education started online; despite the advantages of virtual education, this method cannot replace face-to-face education and only complements face-to-face education [24] and nursing students attend with the direct supervision of clinical instructors in the form of smaller groups to the hospital for internship, but during the internship, students were present in the clinical departments under the indirect supervision of the instructor and the continuous supervision of the clinical supervisor.

Considering the importance of CLE, especially during the pandemic, its impact on the acquisition of professional skills of students and the clinical education experience of the researcher as well as subsequent challenges on the one hand, and the need to investigate the experiences and perceptions of students under these conditions in order to provide useful knowledge in this field on the other hand, the aim of the present qualitative study was to explain the experiences of nursing students in CLEs during the Covid-19 pandemic in order to achieve a deeper and more comprehensive view of the nursing students’ perception.

## Methods

### Design

The present study is a descriptive qualitative study with a contractual content analysis approach, which was conducted between July 2021 and September 2022 to understand the experiences of nursing students in the clinical learning environment during the Covid-19 pandemic. Descriptive qualitative research method is an effective method to understand more about the research question and the participants’ understanding of who, what, the place of the event or their experiences, as well as the understanding and interpretation of the phenomena conducted by the presence of the researcher in the natural environment of the subjects [25]. Qualitative research is considered one of the best research approaches in developing insight and interpretation in the field of nursing [26].

### Participants and setting

The research environment in this study was the Faculty of Nursing and Midwifery affiliated with Shiraz University of Medical Sciences. Fifteen undergraduate nursing students from different academic levels were selected and interviewed through targeted sampling. Sampling in this study continued until data saturation was reached, that is, until no new data was obtained [26].

The inclusion criteria were: internship experience and have entered the clinical learning environment during the covid-19 pandemic, willingness to participate in the study, and the ability to fully express the experiences. The exclusion criterion was the non-cooperation of the participants at any stage of the research.

In the megacities of Iran, such as Shiraz, one or more hospitals were considered as COVID-19 centers, but due to the high prevalence of the disease and the possibility of the capacity of center hospitals being filled, isolation rooms or Covid wards were set up in other hospitals as well. Hospitalized patients should be separated from other patients if they have symptoms of this disease.

### Data collection

To collect information and better understand the experiences of students in the clinical learning environment during the Covid-19, individual and face-to-face interview was used. The interview was conducted at the Faculty of Nursing and Midwifery in person and in a calm environment, with the coordination and willingness of the participants. After explaining the purpose and necessity of the research to the participating students, written informed consent was obtained from them. The participants were explained about maintaining confidentiality and recording their voices during the interview, and if they agreed, their voices would be recorded during the interview.

The interview began with a general and open-ended question, “How did you feel when they told you that you should attend the internship course?“ and continued with more specific questions based on the data from the participants’ statements: “How did you face CLE during the Covid-19 pandemic?“, “What experiences did you have in CLE during this pandemic?“ Attention was gradually focused on the specific issues expressed by the participants, and probing questions such as “Can you explain more?“ or “Give an example” and “How did you feel at that moment?“ were asked.

The duration of each interview was between 30 and 90 min with an average of 60 min. Some participants (4 participants) were interviewed more than once and in multiple sessions. All interviews were transcribed word by word immediately after each session. Sampling continued until data saturation, which was achieved after conducting 15 interviews and analyzing them, when no new information was obtained with the last three interviews and the collected data were indeed repetitions of previous data [25, 27].

### Data analysis

Granheim and Lundman’s content analysis approach approach [28] was used in this study at the same time as data collection, to identify and understand the experiences of nursing students in the clinical learning environment during the Covid-19 pandemic. First, the scripts of the interviews were read several times by the researcher with the aim of being immersed in the study data to achieve a general idea of the interviews. Then, semantic units were extracted from the script of the interviews in the sections related to the experiences of the participants in the clinical learning environment during the Covid-19 pandemic and named in the form of primary codes. It was followed by classification based on similarities and differences between the codes. By considering the internal and external similarity, we tried to distinguish the data in each class in addition to the internal consistency with other classes. Finally, the essences of each of the themes were identified and an attempt was made to gather the meanings of all themes to obtain a comprehensive understanding of the nursing students’ experiences of being placed in the clinical learning environment during the Covid-19 pandemic.

### Trustworthiness

In order to validate the research data, four criteria proposed by Guba and Lincoln were used [25]. After extracting the initial codes, the extracted codes were returned to the participants, and they were asked to comment on the codes. In case of differences between the opinion of the researcher and the participants, the codes were modified. The control on data was conducted by allocating enough time to collect data and getting help from the additional comments of two colleagues familiar with qualitative research on data.

By choosing different participants in terms of age, gender, academic semester, and duration of internship experience during the Covid 19, we tried to consider the transferability of the study results. To ensure the verifiability of the data, the script of a number of interviews, extracted codes, and classes were provided to the researcher’s colleagues and a number of faculty members who were familiar with the method of qualitative research analysis and did not participate in the research. They were asked to examine the accuracy of the data coding process. The research process was also recorded in such a way that other people can follow it by reading the texts.

### Ethical considerations

The Deputy of Research Ethics Committee of Shiraz University of Medical Sciences approved this project prior to the beginning of the study (IR.SUMS.REC.1400.215). In the current study, in order to consider ethical principles, the purpose of the study was explained to all participants and informed consent was obtained for each interview and voice recording. The participants were assured of the data confidentiality. In addition, the recorded interviews were kept in a safe place and were only accessible by the researcher.

## Results

A total of 15 nursing students were recruited into the study. The demographic characteristics of the participants are reported in Table 1.

**Table 1:** Demographic Characteristics of the Participants

Participant	Age (Year)	Gender	Marital Status	Semester
P1	20	Female	Single	4
P2	23	Male	Single	5
P3	25	Female	Married	8
P4	22	Female	Single	8
P5	24	Female	Single	7
P6	25	Male	Single	8
P7	24	Female	Single	7
P8	22	Male	Single	5
P9	23	Female	Single	6
P10	21	Male	Single	4
P11	23	Male	Single	6
P12	24	Female	Single	8
P13	21	Male	Single	7
P14	22	Female	Single	8
P15	21	Female	Single	6

Data analysis led to emergence of two main themes “Disobedience” and “struggle for adaptation” and four categories “objection to being in CLE” (three subcategories), “patient marginalization” (three subcategories), “Using support sources” (three subcategories) and “Using problem-oriented strategies” (three subcategories) (Table 2). “”.

**Table 2:** Themes, Categories and Sub-categories derived from the study

Themes	Main categories	Sub-categories
Disobedience	Objection to being at the bedside Patient marginalization	Being angry at being in CLE Fear of infecting others Bargaining for not holding an internship Escaping from the patient due to ward overcrowding Non-cooperation of the CLE staff due to ward overcrowding Poor patient care
Struggle for adaptation	Use of support sources Applying problem-oriented strategies	Effective interaction Sharing students’ experiences with each other Relying on spirituality Search for information Attention to compliance with protocols Acceptance of the existing situation

### Disobedience

During the covid-19 pandemic, one of the challenges of nursing students in t CLE was the disobedience to attend CLE. The participants, while objecting presence in CLE due to the Covid-19 conditions, always marginalized the patient in the clinical environment. The disobedience consisted of two categories of objecting to being in CLE and the patient marginalization.

#### Objection to being in CLE

The majority of students objected to being in CLE, especially at the onset of the Covid-19 pandemic. These students showed their objection to being in CLE by getting angry, bargaining not to hold an internship, and expressing their fear of getting infected and infecting others.

### Being angry at being in CLE

Some of the participants were angry when they learned about being forced to attend CLE for reasons such as the unknown Covid-19 control measures, the immediate decision of the authorities to hold an internship, ignoring students’ conditions, and concerns by authorities and their families.

*“… when they told me that I should attend the internship, I got angry and started shouting…”* (P.5).

*“… when we found out that the internships were face-to-face, we were all angry, quickly wrote a letter to the Vice-Chancellor of Education on behalf of all students, and objected to attending CLE during this pandemic situation…”* (P. 9).

### Fear of infecting others

According to the participants’ statements, the compulsion to attend CLE had caused them anger, rage, and strong reactions.

Most of the participants were afraid when they were called to attend CLE. They were afraid of getting infected and then infecting others, especially their family members.

*“… Why did we have to get an internship? You know, I was afraid of coronavirus, how can I say, the truth is, I was afraid getting infected, infecting my family, especially my father who had a heart problem…”*(P. 12).

### Bargaining for not holding an internship

After knowing that it is compulsory to attend CLE, most of the participants gathered in the university in order to discuss and convince the authorities to avoid holding internships until obtaining more information on how to control the pandemic, as well as discover vaccines and effective drugs. They submitted their request to the authorities verbally and in writing.

*“…we all gathered in the university, talked with the officials and tried to convince them that now is not the right time to start face-to-internship, but…”* (P. 8).

#### Patient marginalization

Most of the participants believed that the clinical staff did not cooperate with them due to the stressful conditions of CLE; also the ward crowdedness was a factor that made the students anxious and ignore caring for their patients or providing optimal care.

### Non-cooperation of the CLE staff due to ward overcrowding

Due to the crowded and stressful conditions of wards during the Covid-19 pandemic, most of the nurses did not cooperate with the students, did not answer their questions, nor did they inform them about whether the patients were Covid-19 positive or not, which in turn led to an increase in the student’s lack of motivation to provide patient care.

*“… Some days when the ward was overcrowded, the staff didn’t take time or I don’t know, maybe they didn’t care at all to tell us that a certain patient has coronavirus or that we are going to perform the Covid-19 test, and this made use reluctant to take care of the patient under those circumstances "* (P. 7).

### Escaping from the patient due to ward overcrowding

The majority of students did not approach the patients due to the fear of contracting Covid-19 and tried to escape the patients.

*“… On the days when the ward was overcrowded, I didn’t even take the TPR of the patients, because I was afraid to get close to the patients and if did it, I would get more stressed…”* (P.9).

*“… I went to take vital signs of the patient. The patient said: I tested positive and I have corona! I jumped back like when you touch a hot thing. The first warning that came to my mind was to escape him…”* (P. 6).

### Poor patient care

According to the participants, overcrowded/stressful ward conditions during the pandemic led to unfavorable patient care.

*“… When the ward was crowded, I would get more stressed, my self-confidence may decrease. One day, the emergency department was crowded, the professor told me to find that patient’s vein, I was always good at finding veins, but that day I ruptured the poor patient’s vein…”* (P.3).

*“… I could not communicate with the patient like the time when the ward was quiet and there was no coronavirus, or even I could no longer support or take care of patients emotionally when they cried and were upset. I just took a general history and I asked about his symptoms while observing physician distancing distance and went…”* (P. 6).

### The struggle for adaptation

After being forced to attend CLE during the covid-19 pandemic, the students struggled to adapt to the existing conditions through using support sources as well as problem-oriented strategies. Indeed, the struggle for adaptation included two categories of using support sources and using problem-oriented strategies.

#### Using support sources

All participants used various support sources, including recourse and trust in spirituality, effective interaction and experiences of other students, in order to adapt to the Covid-19 conditions in the hospital.

### Effective interaction

All participants talked and shared their experiences with peers, received support from professors, as did well video contact with family members as support sources to adapt to the existing conditions.

*“… I called my faculty advisor and talked about my worries and concerns under this situation. He listened to me patiently, gave me hope and said: Don’t worry. This made me feel a little better…”* (P. 2).

### Sharing students’ experiences with each other

Use of the experiences of other students was another source of support for adapting to CLE during the Covid-19 pandemic.

*“… I sat and talked with the students from other fields or departments who had already gone to the hospital. They were in the Covid-19 center, and told me not to worry at all. We have been following the health protocols for two or three months now, and visiting the Covid-19 patient. We haven’t had any problems and these words have me reassurance…”* (P.6).

### Relying on spirituality

Relying on spirituality made students overcome their fear, adapt to new conditions, and take care of patients.

*“…I prayed a lot and said, “God, when I am observing health protocols, you will definitely not let me get infected. God was my hope, that is, the only thing I could do was to trust God and there is nothing I can do…”* (P.3).

#### Using problem-oriented strategies

The participants coped with covid-19 pandemic using strategies such as searching for covid-19 information, strict adherence to health protocols, and finally accepting the reality and the existing situation by attending CLE.

### Acceptance of the existing situation

All participants attempted to justify themselves and accept the existing conditions in order to attend the clinical environment, considering the uncertainty regarding the end of the pandemic, the possibility of similar pandemics in the future, and choosing the nursing field out of interest and voluntarily.

Acceptance of the existing situation of the Covid-19 pandemic and its consequences was one of the strategies used by all participants in the present study to adapt to the critical conditions.

*“…I said to myself, I am a nursing student, I have already chosen this path, what should I escape from? Is this the last pandemic in our lives! There will definitely be a virus more dangerous than corona in the future, and I have to cope with this issue…”* (P.7).

### Search for information

Considering the unknown nature of the Covid-19 pandemic and the lack of information about it, most of the participants sought to obtain information from up-to-date scientific sources and articles.

One of the participants stated:

*“… I was diligent to access to and read the latest Covid-19 articles and scientific materials. I was searching to see what the latest articles said about manner and duration of washing hands, and used their recommendations…”* (P. 11).

### Attention to compliance with protocols

All participants, upon entering the clinical environment, used their maximum efforts to comply with the health protocols as a strategy to reduce the fear of infection and cope with the Covid-19 conditions.

One of the participants stated:

*“…I tried to be very careful, because the only thing I could do was to wear at least two masks under this situation. I used five-layer masks, changed them every 5–6 hours, washed my hands regularly…”* (P. 1).

## Discussion

The results of the present study revealed that Iranian nursing students experienced disobedience to struggle for adaptation after attending CLE during the Covid-19 pandemic. Disobedience consisted of objecting to presence in CLE and patient marginalization, while struggle for adaptation also involved use of support sources and problem-oriented strategies.

The clinical environment is inherently stressful, and factors such as new situations, changes in the patient’s normal conditions, unfamiliarity with the clinical environment, and working with unpleasant patients cause stress in students [29]. Nursing students are afraid of getting infected and infecting their close family members under critical situations such as a pandemic [22, 30]. Students experience high level of death anxiety when taking care of Covid-19 patients [31]. The fear of getting infected and infecting others, especially parents, was the main reason for the participants’ reluctance to attend the clinical environment. The results of similar studies indicated that students are afraid of getting infected or infecting their relatives [32–35]. The results of Seah et al.‘s study showed that medical and nursing students did not tend to volunteer to attend CLE due to the fear of infecting their family members [35] and even South Korean nursing students decided to leave the nursing profession due to the Covid-19 pandemic [18]. Moreover, nurses often worry about their family getting infected during the outbreak of infectious disease, which is a major barrier to continue their profession [10], while also being afraid to provide care for patients [36]. Overall, fear of infection, lack of personal protective equipment, family opposition or their involvement, fear of infecting family members with the disease, cultural factors, and the death rate caused by the pandemic are among the factors that make students unwilling to voluntarily attend CLE and work during the pandemic [37].

Other studies have shown that the fear of getting infected reduces the interest and enthusiasm towards nursing [38] and this fear was reported as a factor for the unwillingness to take care of patients [39]. Further, students do not wish to work in healthcare centers due to weak control policies during pandemics [40].

The results revealed that increased experience of being in a CLE would lead to reduced stress and enhanced willingness to provide patient care [41]. The results of the Gómez-Ibáñez study showed that senior nursing students volunteered to work in the hospital during the Covid-19 pandemic [42], which is not consistent with the results of the present study. More appropriate personal protective equipment, as well as differences in the number of patients or the ward conditions can be the reasons for the difference in the results of the two studies. In the present study, the participants were nursing students in different years of study, while the participants in Gomez’s study were senior nursing students and had more experience of attending CLE. It was also shown in Collado’s study that although more than 45% of medical and nursing students were afraid of infecting their relatives, this factor did not prevent them from volunteering to attend CLE [43], which is also not consistent with the results of the present study. This difference seems to arise from the intercultural differences and different conditions of Iranian students with the above-mentioned studies in terms of the presence of elderly people or having family members taking immunosuppressive disorders.

Emerging diseases have a negative impact on the behavior and psyche of the general public due to insufficient information about the modes of transmission, the disease course, and the recovery duration [16]. The World Health Organization (WHO) has also emphasized the potential psychological effects of Covid-19 on the general public [44]. There are also negative psychological effects on students due to relatively long periods of quarantine, fear of infection, frustration, impatience, insufficient sources and information, and social stigma [45]. The daily increase in the number of Covid-19 deaths, the news and social media, which are full of Covid-19 discussions, are also effective in this situation and can worsen the existing mental health conditions [9]. In the current study, the students were angry about being called to participate in the internship due to the unknown nature of the coronavirus, the contradictions in and lack of available information on the pandemic control measures, as well as the fear of getting infected and infecting others. Consistent with the results of the present study, Gomez et al.‘s study also reported that nursing students were angry about being in CLEs during the Covid-19 pandemic [42]. The psychological impact of the pandemic on nursing students should not be ignored, and the well-being of these students is affected by high levels of stress and emotion-based coping strategies [30].

Another experience of nursing students from attending CLEs during the pandemic was the crowded and stressful hospital wards, which led to the patient marginalization and providing unfavorable care or escaping from patients. The results of the Ulenaers’s study also showed lack of support from nursing students in CLEs, not inviting students in daily meetings, not informing them about new guidelines, delays in informing students about infected patients and staff. Moreover, half of the nursing students were doubtful about continuing their studies during the pandemic [46]. The results of Godbold’s study also showed that clinical evaluators / supervisors could not devote enough time to students’ learning due to their busy schedule during the Covid-19 pandemic [47]. In a study in Turkey, nurses stated that they did not receive adequate training about Covid-19 and felt that they could not adequately take care of these patients [48].

The participants in the present study used support sources and problem-oriented coping strategies to deal with the stressful pandemic conditions. Searching for updated information about pandemic control measures, effective interaction with others and recourse to spirituality were among the strategies used by the participants in the present study. Adaptation to new conditions is important under crisis situations, especially pandemics, because coping styles help a person adapt to new and challenging situations and stabilize this adaptation [49]. Nursing students as a global group are inherently more vulnerable to psychological stress [50]. Although stress is common among these students, their stress symptoms have become more severe during the Covid-19 pandemic, and these people are struggling to find a healthy stress coping strategy during the pandemic [30]. The results of the above studies, concurring with the present study, suggest that the use of strategies such as searching for information, using spirituality, and family support facilitate adaptation to the Covid-19 pandemic conditions [32, 51, 52]. The results of Sharon et al.‘s study showed that most nursing students during the Covid-19 pandemic used moderate coping strategies [49]. With basic information about the modes of disease transmission, students would change their self-protective behaviors [53]. Savitsky et al. found that searching Covid-19- information is not related to participants’ anxiety level, and information search may increase anxiety during the Covid-19 pandemic, due to the increase of input information, which sometimes spreads contradictory information [22].

Religion helps a person to cope with stressors caused by various crises [54]. The results of Kim et al.‘s study revealed that high resilience, family functioning, and spiritual support are among the factors that reduce stress, anxiety, and depression of nursing students during the Covid-19 pandemic. Also, strengthening these coping mechanisms may help maintain the mental health of students with the pandemic progression [55].

To the author’s best knowledge, this is the first qualitative study that explains nursing students’ experiences of attending CLEs during the Covid-19 pandemic, and provides a deeper and more comprehensive view of nursing students’ perceptions from this environment during the covid-19 pandemic. The results of the present study can thus help managers, planners, and politicians in preparing infrastructure and help students in coping with crises and emerging pandemics. Since the participants of the present study were nursing students, caution must be exercised when generalizing the results to other medical students.

## Conclusion

At the beginning of the pandemic, the students were unfamiliar with the disease, and were afraid of contracting themselves and infecting others, so they tried not to be in the clinical environment. However, they gradually tried to adapt to the existing conditions by applying support resources and using problem-oriented strategies. Policymakers and educational planners can use the results of this study to plan to solve the challenges of students during future pandemics and improve the CLE conditions.

## Data Availability

The datasets generated and analysed during this qualitative study are not publicly available due the ethical reasons but are available from the corresponding author on reasonable request. The informed consent contained a statement that only researchers have access to the raw data and that findings would be presented in an anonymised way.

## Abbreviations

COVID-19: The corona virus disease 2019
CLE: clinical learning environment
WHO: World Health Organization
